# An integrated approach with new strategies for QSAR models and lead optimization

**DOI:** 10.1186/s12864-017-3503-2

**Published:** 2017-03-14

**Authors:** Hui-Hui Hsu, Yen-Chao Hsu, Li-Jen Chang, Jinn-Moon Yang

**Affiliations:** 1 Institute of Bioinformatics and Systems Biology, National Chiao Tung University, Hsinchu, 300 Taiwan; 2 Department of Biological Science and Technology, National Chiao Tung University, Hsinchu, 300 Taiwan

**Keywords:** QSAR model, Computational drug design, Molecular docking

## Abstract

**Background:**

Computational drug design approaches are important for shortening the time and reducing the cost for drug discovery and development. Among these methods, molecular docking and quantitative structure activity relationship (QSAR) play key roles for lead discovery and optimization. Here, we propose an integrated approach with core strategies to identify the protein-ligand hot spots for QSAR models and lead optimization. These core strategies are: 1) to generate both residue-based and atom-based interactions as the features; 2) to identify compound common and specific skeletons; and 3) to infer consensus features for QSAR models.

**Results:**

We evaluated our methods and new strategies on building QSAR models of human acetylcholinesterase (huAChE). The leave-one-out cross validation values *q* ^*2*^ and *r* ^*2*^ of our huAChE QSAR model are 0.82 and 0.78, respectively. The experimental results show that the selected features (resides/atoms) are important for enzymatic functions and stabling the protein structure by forming key interactions (e.g., stack forces and hydrogen bonds) between huAChE and its inhibitors. Finally, we applied our methods to arthrobacter globiformis histamine oxidase (AGHO) which is correlated to heart failure and diabetic.

**Conclusions:**

Based on our AGHO QSAR model, we identified a new substrate verified by bioassay experiments for AGHO. These results show that our methods and new strategies can yield stable and high accuracy QSAR models. We believe that our methods and strategies are useful for discovering new leads and guiding lead optimization in drug discovery.

**Electronic supplementary material:**

The online version of this article (doi:10.1186/s12864-017-3503-2) contains supplementary material, which is available to authorized users.

## Background

As the development in the pharmaceutical chemistry, the computer-aided drug design is a promising direction for shortening the time and reducing the cost for drug discovery. Molecular docking and quantitative structure activity relationship (QSAR) are the important technologies for identifying new leads and lead optimization [1–4]. However, these two methods suffer several challenges: 1) the scoring functions of docking tools are often unable to obtain a high relationship between predicted energies and biological activity values (e.g*.*, binding affinity); 2) docking tools are often designed for one-target paradigm and the scoring methods cannot consistently identify true leads [5]; 3) the performance of 3D QSAR (e.g., CoMFA and COMBINE), highly depends on the superposition of known compound structures and molecular descriptors; 4) the accuracy and selected features of QSAR models are often unstable and lack of biological meanings [1, 4].

To address these issue, we propose an integrated approach by combining in-house tool, GEMDOCK [6, 7], evolutionary algorithms (EAs) [8–10], and partial least square (PLS) regression, with new strategies to construct QSAR models for discovering new leads and guiding lead optimization. GEMDOCK has yielded comparable molecular docking and screening performance to other docking tools, such as FlexX and GOLD [6]. In addition, GEMDOCK has been successfully applied to the discovery of novel inhibitors and binding mechanisms for some target proteins [4, 11–14]. Here, we applied genetic algorithms (GAs) [10], simulating the natural selection mechanisms, and PLS regression, which is a simple statistical method, to select the key features (i.e., core functional groups of inhibitors) from protein-ligand interactions for improving accuracies of QSAR methods.

To infer the protein-ligand interaction for QSAR and lead optimization, we have developed three core strategies. First, we used GEMDOCK to predict protein-ligand complexes for generating residue-based and atom-based interaction profiles as the features of QSAR models. Second, we statistically inferred consensus features from the selected interactions of preliminary QSAR models built by GEMPLS and GEMkNN. These consensus features are often able to reflect biological meanings, such as the key residues for evolutionary conservation, protein functions, and ligand binding. Third, we identified common/specific skeletons of the inhibitors for lead discovery and optimization. In general, the common skeletons, highly shared by the inhibitors of a target protein, form the basic scaffolds to interact with key residues which often occupy the critical pocket of a target protein. Conversely, the specific skeletons, which are the substitution function groups occupying specific subsites, of these inhibitors can be used for lead optimization to increase the potency.

We evaluated our method and new strategies on QSAR models of human acetylcholinesterase (huAChE), which is one of promising therapeutic targets for central nervous system diseases, such as Alzheimer's disease [15]. The *q* ^*2*^ and *r* ^*2*^ values of our huAChE QSAR model are 0.82 and 0.78, respectively. In addition, the selected features (resides/atoms), forming key interactions with its inhibitors, play the key role for protein functions and structures. Furthermore, we applied our method to arthrobacter globiformis histamine oxidase (AGHO), which is important for metabolisms of biogenic primary amines and is correlated to heart failure [16] and diabetic patients [17, 18]. Using our QSAR model, we identified a new substrate evaluated by bioassay experiments. We believe that our methods and strategies are useful for building QSAR models, discovering leads, and guiding lead optimization.

## Methods

### huAChE and AGHO

Acetylcholinesterase (AChE, carboxylesterase family of enzymes) catalyzes the hydrolysis of acetylcholine (ACh) in cholinergic synapses which are important for neuromuscular junctions and neurotransmission. To evaluate our method and compare with other methods, we collected 69 inhibitors with IC_50_ of huAChE from previous work [19], which divided the set into the train set (53 inhibitors, Additional file 1: Table S1) and testing set (16 inhibitors, Additional file 2: Table S2). In addition, we applied our methods to AGHO, which is the member of CuAOs family, to construct its QSAR model. Based on our model, we identified a new substrate of AGHO and verified by bioassay experiments.

### Overview for building QSAR models

**Fig. 1:**
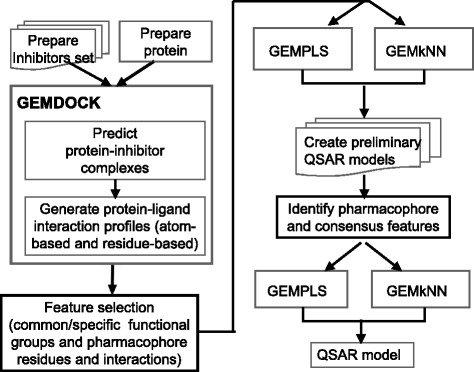
The main steps of our method. For a target protein, we first use in-house docking tool, GEMDOCK, to identify the potential leads with protein-lead complex and generate protein-lead interaction profiles used as the QSAR features. GEMPLS and GEMkNN are applied for feature selection and building preliminary QSAR models to statistically yield the consensus features. Based on known lead structures and consensus interaction features, we infer the ligand common/specific skeletons to construct robust QSAR models and lead optimization

We integrated GEMDOCK with GEMPLS/GEMkNN and common protein-ligand interactions (considered as the hot spots of a target protein) for building QSAR modeling (Fig. 1). To identify the protein-ligand interactions for QSAR model, we developed three strategies: i) use both residue-based and atom-based as the QSAR features; ii) inferring consensus features from preliminary QSAR models; iii) identifying compound common/specific skeletons from the compound set. Based on these strategies, our method yielded a stable QSAR model which is able to reflect biological meanings and guide lead optimization. The main steps of our method are described as follows: 1) prepare the binding site of the target protein; 2) prepare and optimize compound structures using CORINA3.0 [20]; 3) predict protein-compound complexes and generate atom-based and residue-based interactions using GEMEDOCK; 4) identify common/specific ligand skeletons by compound structure alignment; 5) create *N* (here, *N* = 30) preliminary QSAR models using GEMPLS and GEMkNN based on leave-one-out cross validation; 6) statistically identify consensus features using these preliminary QSAR models; 7) create QSAR models using GEMPLS and GEMkNN with consensus/specific features. The leave-one-out cross validation uses one inhibitor as the test set and the remaining inhibitors as the training set, and this procedure is repeated *n* times, where *n* is the number of inhibitors.

### GEMDOCK and interaction profiles

Here, we briefly described GEMDOCK for molecular docking and generating atom-based and residue-based interactions. For each inhibitor in the data set, we first used GEMDOCK to dock all inhibitors (Additional file 1: Table S1) into the binding site of target protein (huAChE). GEMDOCK is an in-house molecular docking program using piecewise linear potential (PLP) to measure intermolecular potential energy between proteins and compounds [6]. GEMDOCK has been successfully applied to identify novel inhibitors and binding sites for some targets [4, 11–14]. The PLP is a simple scoring function and is comparable to some scoring functions for estimating binding affinities [21–23].

**Fig. 2:**
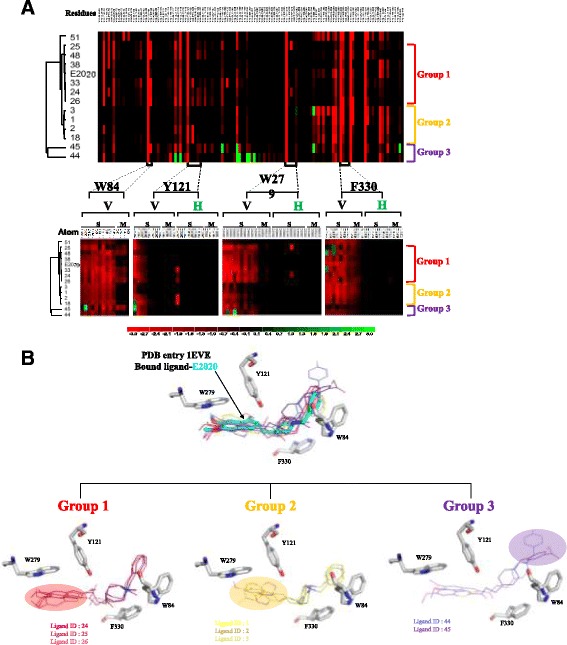
Interaction profiles and typical docked poses of tcAChE using GEMDOCK. **a** The atom-based and residue-based features of 14 docked inhibitors of tcAChE (PDB entry 1EVE) using GEMDOCK. These 14 inhibitors can be clustered into 3 groups based these features. **b** Typical docked poses of these three groups, including Group 1 (Ligands 24, 25, and), Group 2 (Ligands 1, 2, and 3), and Group 3 (Ligands 44 and 45). The structures and Ligand IDs of these 8 inhibitors are shown in Additional file 1: Table S1

Based on these docked poses, we used GEMDOCK to generate both residues-based and atom-based interaction as the features. The atom-based and residue-based interaction profiles of target-compound complexes were extracted by applying this PLP. The profiles include electrostatic, hydrogen-bonding, and van der Waals interactions between the compounds and the protein. Figure 2a shows 2118 atom-based features and 516 residues-based features on 86 residues for 14 inhibitors. Some residue features, such as VDW force (V) with side chain (S) of W84, Y121, W279 and F330, of these 14 docked poses were consensus with tcAChe bounded ligand E2020. According to the residue-based and atom-based features, we can cluster these 14 compounds into three groups (Fig. 2b). We show several representatives of docked compounds in each group and the docked compounds in the same group contain the common skeletons.

The inhibitors with the reference bounded ligand E2020 in the group 1 form similar residue-based and atom-based features. The residue-based and atom-based features of inhibitors in the group 2 have significantly different in main chain (M) of W84 and side chain (S) of W279 from the ones of inhibitors in group 1. In addition, we can find that the common skeletons of the inhibitors in group 2 (yellow circles) are different from the ones of inhibitors in group 1 (red circle). The group-2 inhibitors contain the N atom in bicyclic (yellow circle), but group-1 inhibitors loss the N atom in bicyclic and connect the O atom (red circle) (Fig. 2b and Additional file 1: Table S1). The common skeletons of inhibitors in group 3 are different with the other groups. The skeleton (purple circle), which forms the repulsive forces, of group-3 inhibitors is longer than the ones of other groups. GEMDOCK and the datasets are freely accessed at http://gemdock.life.nctu.edu.tw/dock/download.php.

#### Modeling active form structure of huAChE

**Fig. 3:**
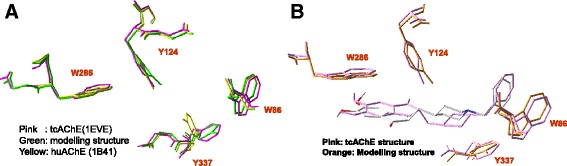
Structure simulation and docked pose of huAChE. **a** The structural alignment of huAChE (PDB entry 1B41, yellow), tcAChE (PDB entry 1EVE with bounded ligand E2020, pink) and modeled structures (green). The main difference among the three structures is the side-chain conformation of Y337 (residue number of 1B41, relatively to F330 in 1EVE). In the structure of 1EVE, the F330 was in active-form position to stabilize the ligand and protein-ligand complex. The structure of 1B41 is a close form and Y337 is not an active-form conformation. The conformation of Y337 is in the active position in the modeling structure. **b** The docked pose (white) of E2020 is similar to the position of the ligand (pink) in tcAChE x-ray structure (1EVE).ing materials

The availability of protein X-ray structure with suitable induced form is important for molecular docking and QSAR. We observed the structures of human AChE (huAChE, PDB entry 1B41 [24], without ligand-bounded complex) and *Torpedo californica* AChE (tcAChE, PDB entry is 1EVE [25]) with ligand (called E2020) co-crystallized complex by aligning these two structures with maximal overlap of Cα atoms. The sequence identity is 57% and the root mean square deviation (RMSD) value of these two structures is 0.88 Å (Fig. 3). We found these two structures with significant conformation change for the residue Y337 (residue number in huAChE, Fig. 3a), which is the gate for protein function and inhibitor binding [25–27]. Therefore, we used both huAChE and tcAChE to model the active-form conformation of huAChE. We first constructed the huAChE-E2020 complex by inserting E2020 into huAChE structure. We then added hydrogen atoms to this huAChE-E2020 complex and minimized this structure by using SYBYL7.0 modeling software package. Finally, we used this minimized huAChE-E2020 complex for the molecular docking and QSAR model.

#### GEMPLS (GEM-partial least squares)

GEMPLS is a QSAR method by combining the statistical method (PLS-regression) and Gas which select the features from protein-ligand interactions. In GEMPLS, the chromosomes consist of randomly selected features and the latent variable (*lv*), which appends to the chromosome to find optimum number of latent variables. The squared cross-validated correlation coefficient *q* ^*2*^ in the PLS analysis is used as the objective function measuring the performance using the selected features of the chromosome. GA selected the features with the highest *q* ^*2*^ in the PLS analysis. To improve the performance of GEMPLS for QSAR model building, we adopt Mahalanobis distance to discriminate significant features and designed a biased genetic mutation, that is, the significant feature *i* with higher Mahalanobis distance will get the higher *P*_*i*_, to replace the uniform mutation. Here, Mahalanobis distance is define as *M* ^2^ = (*v* − μ) ' ∑^− 1^(*v* − μ), where *M* is the Mahalanobis distance from the feature vector *v* to the mean vector *μ*, Σ is the covariance matrix of the features. We set the threshold of Mahalanobis distance to 10 by testing our method with various threshold values for achieving optimal performance. Experimental results show that Mahalanobis distance can successfully discriminate significant features and reduce the ill effect of selecting numerous selected features.

#### GEMkNN (GEM- k-nearest-neighbor)

GEMkNN integrated GAs and kNN, which is a conceptually simple and nonlinear approach. GEMkNN is similar to GEMPLS and the main difference is that we used the k-nearest neighbor algorithm to replace PLS. In GEMkNN, the chromosomes consist of randomly selected features and the number of *k* nearest compounds. The similarities between the compounds are evaluated by Mahalanobis distance. The squared cross-validated correlation coefficient *q* ^*2*^ in the kNN analysis is used as the objective function of GAs.

#### Identifications of consensus features

**Fig. 4:**
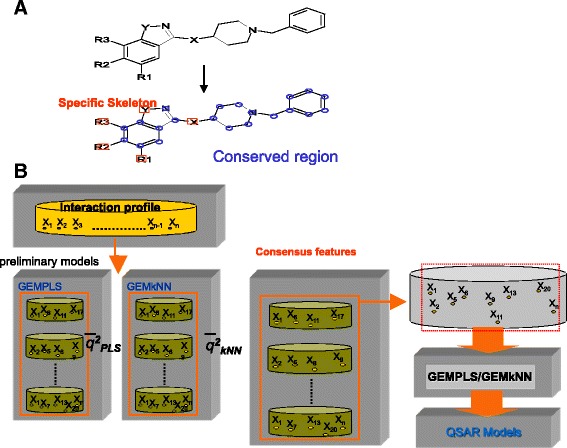
Core strategies for the protein-ligand interaction hot spots. **a** Identify common/specific skeleton. The skeletons (*blue circles*) highly shared by most inhibitors of a target protein is called common skeleton, the other parts are called specific skeletons (*red squares*). In general, the common skeletons often form consensus interactions with key residues of the target protein and specific skeletons are often the substitution function groups (e.g., R1, R2, R3, X, and Y) occupying specific functional subsites for lead optimization to increase the potency. **b** Infer consensus features from preliminary QSAR models. Based on the selected features in preliminary QSAR models, we count the selected times (*N*_*i*_) for each feature *i* and calculate the average (μ) and standard deviation (σ) based on all features. The feature *i* is selected as consensus feature candidate if its *N*_*i*_ ≥ (μ-σ)

For building QSAR models, we found that the performance and the selected features of both GEMPLS and GEMkNN are often unstable when the number of features is over 100. To address this issue, we proposed the strategy to select consensus features by executing both GEMPLS and GEMkNN 30 times (Fig. 4). Based on the selected features in these 60 times, we counted the selected times (*N*_*i*_) for each feature *i*, and then calculated the average (μ) and standard deviation (σ) of all features. Furthermore, we selected the feature *i* as the candidate feature if its *N*_*i*_ ≥ (μ-σ) and these features are considered as the consensus feature candidates (e.g., *X*_*1*_, *X*_*2*_, and *X*_*6*_ in Fig. 4b). Finally, GEMkNN and GEMPLS employed these consensus feature candidates as descriptors to construct the QSAR models.

#### Performance evaluation

We evaluated the accuracies of QSAR models using cross-validated correlation coefficient (*q* ^*2*^) and the standard deviation of error of prediction (*SDEP*). They are defined as follows:

$$ {q}^2=1-\frac{\sum {\left({y}_i-{y}_{pred, i}\right)}^2}{\sum {\left({y}_i-\overline{y}\right)}^2} $$ and $$ SDEP=\sqrt{\frac{\sum {\left({y}_i-{y}_{pred, i}\right)}^2}{N}} $$

where *y*_*i*_ and *y*_*pred,i*_ are the experimental and predicted activities, respectively, of the compound *I*; $\overline{y}$ and *N* are the average biological activities and total number of inhibitors in the data set, respectively. The highest *q* ^2^ and lowest *SDEP* can be used to assess the predictability of a QSAR model.

## Results and Discussions

### Modeling structure of huAChE and evaluation GEMDOCK on AChE

Figure 2a shows the structures of the modeled (green) and crystal structures of huAChE (1B41, yellow) and tcAChE (1EVE, pink). The RMSD values are 0.24 Å and 0.89 Å between modeled structure and 1B41 and 1EVE, respectively. In the active site, the main difference among the three structures is the side-chain conformation of Y337 (residue number in huAChE, related to F330 in tcAChE). The complex structure of tcAChE shows that the residue F330 is induced into a wide range of conformations and plays a key role as the gate for protein function and inhibitor binding [25–27]. This result implies that Y337 in huAChE should be flexible when the protein binds with different inhibitors. In addition, most docked poses of huAChE inhibitors were not correct to form the stack force with Y337 using the crystal structure (1B41) of huAChE. Based on these results, we used both crystal structures of huAChE and tcAChE (1EVE) to model the bounded structure of huAChE to yield the induced conformation of Y337.

To evaluate GEMDOCK, we first docked the bounded ligand (E2020) into the binding sites of target protein (tcAChE, 1EVE) and the modeled huAChE (Fig. 2). Here, we defined that the binding site is the amino acids enclosed within a radius of 8 Å relative to the E2020. The RMSD of the ligand (E2020) between the predicted (white) and x-ray structure (pink, tcAChE) is 1.73 Å. this docked pose forms a stable stack force with W84, W279 and F330 with target proteins. In addition, for the modeled huAChE, most docked poses of these 69 huAChE inhibitors in the data set form stable forces with residues W86, W286, and Y337. These results show that GEMDOCK is able to generate reasonable docked poses for huAChE and the modeled huAChE structures.

### Evaluation QSAR models on huAChE

We evaluated our QSAR methods on huAChE with a public compound set [19]. The compound set includes 69 compounds with IC_50_ values and those ligands can be grouped into four kinds of derivative. To compare our method with previous method (Table 1), we used the same performance metrics and data sets, including training set (53 compounds) and testing set (13 compounds) [19]. The IC_50_ values of ligands range from 0.48 *nM* to 19,580 *nM* in the training set and 0.33 *nM* to 30,000 *nM* in the testing set. To evaluate the core strategies of the protein-ligand hot spots for QSAR models, we tested our methods on all interaction features, the consensus features statistically inferred from preliminary QSAR models, and the features of specific skeletons (i.e., substitution function groups by discarding common skeleton) (Table 2). For example, the total numbers of the features are 223 (all features) and 156 (consensus features) if we used atom-based interaction profiles. Our GEMPLS method selected 36.2 and 22 features, respectively, if we applied all interactions and consensus interactions as the QSAR features (Table 2). In addition, our QSAR method selected 10 interaction residues in huAChE if we used the residue-based features (Table 3). These selected residues were consensus interactions and some residues play the key roles for the catalytic triad in protein active site.

**Table 1:** Comparisons our method with Guo’ method

	Our method	Guo et al.
Docking Tool	GEMDOCK	GOLD
Features	Atom/residue features	Residue features
*q* ^*2*a^	0.82 (mean)	0.72
*r* ^*2*^ ^b^	0.723 (mean)	0.63

^a,b^The *q* ^*2*^ and *r* ^*2*^ values are the means of 30 independent QSAR models

**Table 2:** Accuracies of our method using different protein-ligand interaction profiles on huAChE set

	All interaction profile^a^		Consensus feature profile^b^		
	GEMPLS	GEMkNN	GEMPLS	GEMkNN	Specific^g^ Skeleton
No. of total features (atoms)	223	223	78	156	92
No. of selected features (atoms)	36.2	36.1	22	29.8	23.5
Average of *q* ^*2*^ (Training)^c^	0.627	0.657	0.658	0.704	0.817
Average of *r* ^*2*^ (Testing)^d^	0.402	0.123	0.467	0.063	0.723
Standard derivation of *q* ^*2e*^	0.015	0.018	0.004	0.009	0.006
Standard derivation of *r* ^*2f*^	0.125	0.095	0.085	0.050	0.056

^a,b^Using all and only consensus interaction profiles as descriptions, respectively

^c,d^The average *q* ^*2*^ and *r* ^*2*^ values of 30 times for the training set and testing set, respectively

^e,f^The standard deviation *q* ^*2*^ and *r* ^*2*^ values of 30 times for the training set and testing set, respectively

^g^Using the interaction profiles of both consensus feature profile and specific skeleton

**Table 3:** Selected residues and biological meanings of huAChE QSAR model

Selected residue	Descriptions
TYR72	Stabilize ligand ring
TRP86	Forming π-π interaction to stabilize ligand
ASN87	Electrostatic contributors in the gorge area
TYR124	Form hydrophobic contacts with ligand
SER203	Catalytic triad
TRP286	Enhance the activity of ligand with polar groups
TYR337	Electrostatic contributors in the gorge area
PHE338	Form hydrophobic contacts with ligand
TYR341	The residue in the local pocket
HIS447	Catalytic triad

The common metrics were used to evaluate the quality of QSAR models, including the *q* ^*2*^ (cross-validated correlation coefficient) in the training set and *r* ^*2*^ (correlation coefficient) in the testing set. To validate the stability of the method for QSAR models, we built 30 models for each kind of conditions and then evaluated the mean and standard deviation values of *q* ^*2*^ and *r* ^*2*^. Table 1 shows the comparisons of our method with Guo et al.’ method [19] in which they used GOLD for docking simulation and residue-based descriptions. For Guo’ method, the *q* ^*2*^ value is 0.72 and the *r* ^*2*^ value is 0.69. Our method is very comparative to their method and the *q* ^*2*^ and *r* ^*2*^ values are 0.81 and 0.72, respectively, on the same data set.

The *q* ^*2*^ values of GEMPLS and GEMkNN using all features (0.627 and 0.657, respectively) and consensus interaction features (0.658 and 0.704, respectively) are comparable (Table 2). For the *r* ^*2*^ value, the GEMPLS outperformed GEMkNN for this set. These two methods consistently yielded stable accuracies when they used consensus interaction features as the descriptors. In addition, experimental results show that our methods can achieve the best *q* ^*2*^ (0.817) and *r* ^*2*^ (0.723) values by using both consensus and specific features by discarding the features of common skeletons (Table 2).

To evaluate the biological meanings of our QSAR, we observed the protein-compound complexes to check the selected features/residues (Table 3). For example, the residues S203 and H447 are catalytic triad in huAChE, and the residue Y72 forms a wall to stabilize ligand and W86 forms π-π interaction with choline. In addition, the residues N87 and Y337 contribute the electrostatic force in the active site, residue W286 affects the binding affinity of AChE inhibitors, and residues Y124 and F338 provide hydrophobic contacts with bounded ligands. These results reveal that our method is able to achieve high accuracy QSAR models and reflect the biological and structure meanings.

### Common/specific skeletons of ligands

In general, the inhibitors of a target protein share highly common skeletons (blue circles in Fig. 3a, Additional file 1: Table S1, and Additional file 2: Table S2), which form key interactions with key residues in the binding site of the protein. For lead optimization, the substituent functional groups (red squares, e.g., R1, R2, X, and Y in Fig. 3a) on the branch of the common skeletons (blue circles) are key to decide biological activities and potency of these derivatives. However, these substituent functional groups are often a small proportion in a ligand. To enhance the accuracy of QSAR models and lead optimization, we concentrated on features of the substituent groups by discarding the features of common skeletons.

### Real case study on AGHO

**Fig. 5:**
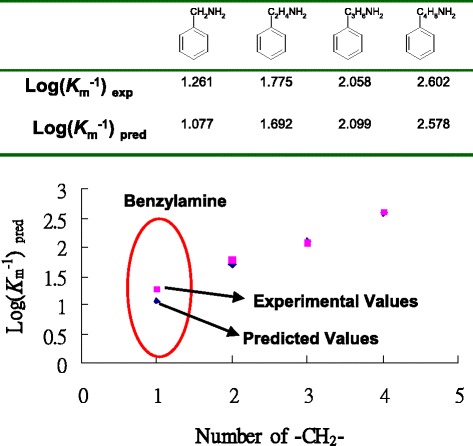
The relationship between experimental values and predicted affinities of AGHO QSAR model. The side-chain length of compound is highly correlated to the compound affinities. In addition, we discover the benzylamine as potential inhibitors and the predicted affinity is 1.077 and the experimental bioassay value is 1.261

We have applied our methods to construct the QSAR model of AGHO, which is the member of CuAOs family (Fig. 5). Based on our QSAR model, we identified a new AGHO inhibitor. CuAOs are important for ubiquitous and have a variety of function in the metabolism of biogenic primary amines. Here, we do not show the details of the modelling AGHO structures, collecting compound data set, generating protein-inhibitor interactions, constructing QSAR models, and the bioassay assays.

Our results show that the side-chain length of substrates is highly correlated to the compound affinity (Fig. 5). The predicted IC50 values of our QSAR model increase with the increase of side-chain length and are highly related to real experimental test values. This tendency is similar to the phenomenon among phenylethylamine, phenylpropylamine and phenylbutylamine. We used GEMDOCK to screen the similar compounds of phenylethylamine from a public database. We discovered the benzylamine as a potential substrate and then utilized our QSAR model to predict its affinity (1.077) which is similar to experimental bioassay value (1.261) (Fig. 5). This result suggests that the hydrophobicity is one of the essential factors (common skeleton) and the side-chain length (specific skeletons) determines the affinity for AGHO. In addition, our QSAR selected some important residues, such as Asp 317 is the proton acceptor of AGHO, the residues Ala 155, Pro 156, Tyr315 and Phe 426 forming the hydrophobic pocket [28]. These experimental results show that our method can build the QSAR models of AGHO for discovering new substrates and revealing biological meanings.

## Conclusion

We have integrated in-house tools, GEMDOCK and GEMPLS, with new strategies to identify the protein-ligand hot spots for building QSAR models, lead discovery and optimization. Experimental results show that these strategies are able to enhance the advantages of virtual screening and QSAR by identifying the consensus interaction profiles and common skeletons. In addition, the specific skeletons of compounds are useful for lead optimization and improving the performance of QSAR models by reducing the number of features. Furthermore, the selected features of QSAR models provide the clues for protein functions and binding mechanisms. Our methods and strategies successfully discovered a new substrate for AGHO. These results demonstrate that our methods with new strategies are useful to yield stable and high accuracy QSAR models for discovering new leads and guiding lead optimization.

## Additional files

Additional file 1: Table S1. Compound training set for huAChE collected from Guo et al. (PDF 219 kb)

Additional file 2: Table S2. Compound testing set for huAChE collected from Guo et al. (PDF 52 kb)
