# Supplementary material for: An integrated approach with new strategies for QSAR models and lead optimization
Source: BMC Genomics. 2017 Mar 14;18(Suppl 2):104. doi: 10.1186/s12864-017-3503-2 (PMC5374651; doi:10.1186/s12864-017-3503-2)
Supplement: Supplementary file 1 — Compound training set for huAChE collected from Guo et al. (PDF 219 kb) [file 12864_2017_3503_MOESM1_ESM.pdf]

Table S1. **Compound training set for huAChE collected from Guo *et al*.**

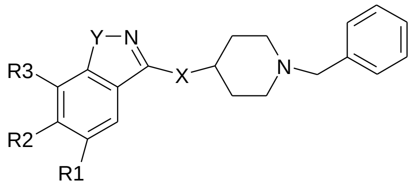

R1	R2	R3	-X-	-Y-	Ligand ID	IC50(nM)	pIC_{50}
-H	-H	-H	$-(CH_2)_2-$	-O-	1	55	7.26
$-CH_3$	-H	-H	$-(CH_2)_2-$	-O-	2	7.8	8.11
$-CH_3$	$-OCH_3$	-H	$-(CH_2)_2-$	-O-	3	5.8	8.24
$-OCH_3$	-H	-H	$-(CH_2)_2-$	-O-	4	7.2	8.14
-H	-H	$-OCH_3$	$-(CH_2)_2-$	-O-	5	7.1	8.15
-H	$-NH-CO-CH_3$	-H	$-(CH_2)_2-$	-O-	6	2.8	8.55
-H	-NH-SO2-Φ	-H	$-(CH_2)_2-$	-O-	7	14	7.85
-H	-4-morpholino	-H	$-(CH_2)_2-$	-O-	8	0.8	9.10
-H	$-NH_2$	-H	$-(CH_2)_2-$	-O-	9	20	7.70
-H	-Br	-H	$-(CH_2)_2-$	-O-	10	50	7.30
-H	-CN	-H	$-(CH_2)_2-$	-O-	11	101	7.00
-H	$-CO-NH_2$	-H	$-(CH_2)_2-$	-O-	12	8.8	8.06
-H	-H	-H	$-(CH_2)_3-$	-O-	13	900	6.05
-H	-H	-H	$-O-CH_2-$	-O-	14	2600	5.59
-H	-H	-H	$-NH-CH_2-$	-O-	15	320	6.49
-H	-H	-H	$-(CH_2)_2-$	-S-	16	99	7.00
-H	-H	-H	$-(CH_2)_2-$	-CH=CH-	17	220	6.66
-H	-H	-H	$-(CH_2)_2-$	-NH-	18	120	6.92
$-CH_2-CH_2-CO-NH-$		-H	$-(CH_2)_2-$	-O-	19	0.57	9.24
$-NH-CO-CH_2-$		-H	$-(CH_2)_2-$	-O-	20	0.95	9.02
$-N(CH_3)-CO-CH_2-$		-H	$-(CH_2)_2-$	-O-	21	0.48	9.32
-H	$-NH-CO-CH_2-$		$-(CH_2)_2-$	-O-	22	3.6	8.44
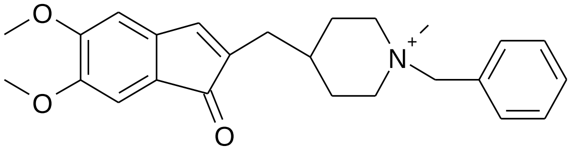					23	250	6.60

Table S1. Continued

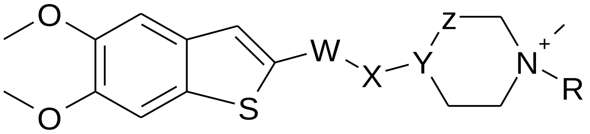

-W-	-X-	-Y-Z-	R	Ligand ID	IC50 (nM)	pIC_{50}
-(CO)-	$-CH_2-CH_2-$	$-CH-CH_2-$	-CH2-Φ	24	8	8.10
-(CO)-	-CH2-C(OH)-	$-CH-CH_2-$	-CH2-Φ	25	43	7.37
-(CO)-	$-CH_2C(OH)CH_2CH_2-$	$-CH-CH_2-$	$-CH_2$-Φ	26	380	6.42
-(CO)-	$-CH_2CH_2CH_2CH_2-$	$-CH-CH_2-$	$-CH_2$-Φ	27	110	6.96
-(CO)-	$-CH_2C(OCH_3)-$	$-CH-CH_2-$	$-CH_2$-Φ	28	120	6.92
-(CO)-	-CH-	$-C-CH_2-$	$-CH_2$-Φ	29	520	6.28
-C(OH)-	-	$-CH-CH_2-$	$-CH_2$-Φ	30	19580	4.71
-	-CH-	$-C-CH_2-$	$-CH_2$-Φ	31	2670	5.57
-(CO)-	$-CH_2-CH_2-$	$-CH-CH_2-$	$-(CH_2)_2OCH_3$	32	53	7.28
-(CO)-	$-CH_2-CH_2-$	$-CH-CH_2-$	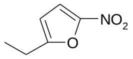	33	32	7.49
-(CO)-	$-CH_2-CH_2-$	$-CH-CH_2-$	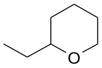	34	28	7.55
-(CO)-	$-CH_2-CH_2-$	$-CH-CH_2-$	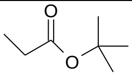	35	79	7.10
-(CO)-	$-CH_2-CH_2-$	$-CH-CH_2-$	$-CH_2CH_2$-O-Φ	36	390	6.41
-(CO)-	$-CH_2-CH_2-$	$-CH-CH_2-$	$-CH_2$-CN	37	1000	6.00

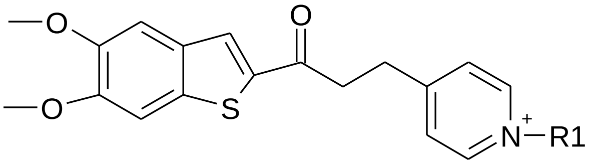

-R1	Ligand ID	IC50(nM)	pIC_{50}
$-CH_3$	38	900	6.05
$-CH_2CH_3$	39	280	6.55
$-CH_2CH=CH_2$	40	540	6.27
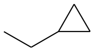	41	110	6.96
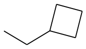	42	40	7.40

Table S1. **Continued**

**-R1**	**Ligand ID**	**IC50(nM)**	**pIC_{50}**
$-CH_2CH_2-O-CH_2CH_3$	43	7	8.15
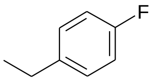	44	2.6	8.59
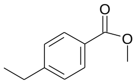	45	1000	6.00
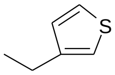	46	6	8.22
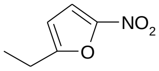	47	4.5	8.35
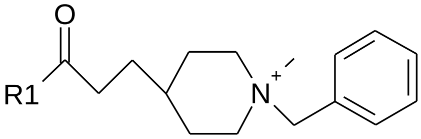			
**-R1**	**Ligand ID**	**IC50(nM)**	**pIC_{50}**
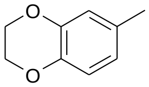	48	100	7.00
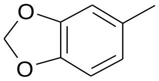	49	41.5	7.38
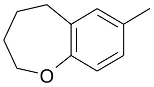	50	139	6.86
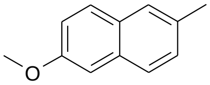	51	50	7.30
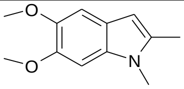	52	120	6.92
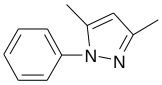	53	22	7.66
