# Supplementary material for: An integrated approach with new strategies for QSAR models and lead optimization
Source: BMC Genomics. 2017 Mar 14;18(Suppl 2):104. doi: 10.1186/s12864-017-3503-2 (PMC5374651; doi:10.1186/s12864-017-3503-2)
Supplement: Supplementary file 2 — Compound testing set for huAChE collected from Guo et al. (PDF 52 kb) [file 12864_2017_3503_MOESM2_ESM.pdf]

Table S2. Compound testing set for huAChE collected from Guo et al.

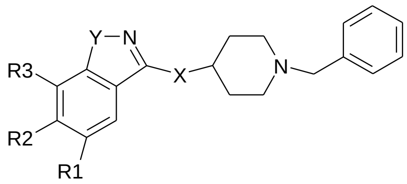

R1	R2	R3	-X-	-Y-	Ligand ID	IC50(nM)	pIC_{50}
-H	$-OCH_3$	-H	$-(CH_2)_2-$	-O-	54	8.3	8.08
-H	-NH-CO-Φ	-H	$-(CH_2)_2-$	-O-	55	9.4	8.03
-H	-OH	-H	$-(CH_2)_2-$	-O-	56	26	7.59
-H	-H	-H	$-(CH)_2-$	-O-	57	210	6.68
-H	-H	-H	$-NH-(CH_2)_2-$	-O-	58	810	6.09
-H	-H	-H	$-(CH_2)_2-$	$-N=CH_2$	59	340	6.47
$-CH_2CONH-$		-H	$-(CH_2)_2-$	-O-	60	0.33	9.48

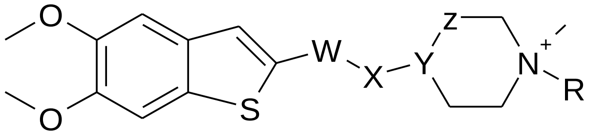

-W-	-X-	-Y-Z-	R	Ligand ID	IC50(nM)	pIC_{50}
-(CO)-	$-CH_2C(OH)-$	$-CH-CH_2-$	-CH2-Φ	61	190	6.72
-(CO)-	$-CH_2-$	$-C(OH)-CH_2-$	-CH2-Φ	62	90	7.05
-(CO)-	$-CH_2-$	-C=CH-	-CH2-Φ	63	750	6.12
-	$-CH_2-$	$-CH-CH_2-$	-CH2-Φ	64	30000	4.52
-(CO)-	$-CH_2CH_2-$	$-CH-CH_2-$	$-CH_2COOCH_3$	65	54	7.27

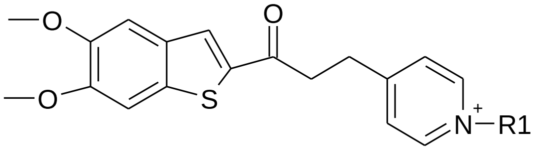

-R1	Ligand ID	IC50(nM)	pIC_{50}
$-(CH_2)_2CH_3$	66	2570	5.59
$-(CH_2)_2OCH_3$	67	30	7.52
-CH2-Φ	68	4.6	8.34
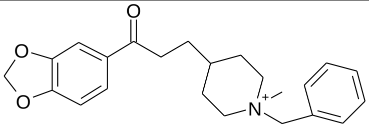	69	240	6.62
